# Reduction in live births in Japan nine months after the Fukushima nuclear accident: An observational study

**DOI:** 10.1371/journal.pone.0242938

**Published:** 2021-02-25

**Authors:** Alfred Körblein

**Affiliations:** Untere Söldnersgasse 8, Nürnberg, Germany; University of South Carolina, UNITED STATES

## Abstract

**Background:**

After the Chernobyl accident on 26 April 1986, a drop in birth-rate was found in several European countries in the first quarter of 1987. The objective of the present study was to investigate whether a similar drop in live births occurred in Japan after the Fukushima nuclear accident.

**Data and methods:**

A study region was defined consisting of Fukushima prefecture plus 10 nearby prefectures. The observed monthly numbers of live births (LB) in October 2011 through December 2012 were compared with the predicted numbers determined from the trend of live births in the remaining months from January 2006 through December 2018. The study region was divided into Fukushima plus three adjacent prefectures (Area A, assumed effective mean dose in the first year 1 mSv) and seven surrounding prefectures (Area B, 0.5 mSv). The rest of Japan (Area C) served as the comparison (control) region (0.1 mSv). A combined regression of live births (LB) in areas A, B, C was conducted with individual trend parameters but common parameters for monthly variations.

**Results:**

In the study region as a whole (areas A and B combined) a highly significant 9.1% (95% CI: -12.2%, -6.0%) drop in LB was found in December 2011. Reduced numbers of live births were also observed in October-November 2011 (-3.3%, p *=* 0.006), i.e. in births exposed early in pregnancy. In the second quarter of 2012, i.e. in live births conceived more than 3 months after the Fukushima accident, the decrease was greater (-4.3%, p *<* 0.001) than in the first quarter (-1.6%, p = 0.11). i.e. in those conceived within the first three months after the accident while no significant decrease was detected in the third (-0.7%, p = 0.44) and fourth (-0.5%, p = 0.62) quarters. The effect in Dec 2011 was greater in Area A with -14.0 (-17.6, -10.3) % than in Area B with -7.8 (-11.1, -4.5) % and non-significant in Area C with -1.3 (-4.2, +1.6) %, p = 0.38. The combined regression of the data in areas A, B, and C found a highly significant association of the effect in December 2011 with radiation dose. Conclusion: It is suggested that the observed drop in LB in December 2011 may reflect early deaths of the conceptus from high radiation exposure following the triple meltdown at the Fukushima Daiichi nuclear power plant on March 12–15, 2011.

## Introduction

After the Chernobyl nuclear accident on 26 April 1986, a drop in birth rates was found in the first three months of 1987 (i.e. 9 ± 1 months after the accident), in several European countries, including Greece [1], Italy [2], Sweden [3, 4], Norway [5], Finland [6, 7], and Hungary [8]. The observed decrease in live births (LB) was generally attributed to anxiety. Källen [4] explained the effect by “public scare after the accident, often supported by the media”. And Auvinen et al. [7] stated: “The decrease in the live birth rate is probably not a biological effect of radiation, but more likely related to public concerns of the fallout."

Studies of LB were also conducted in Japan after the Fukushima nuclear accident on 12–15 March 2011 [9–11]. Kurita [11] studied birth rates in Fukushima City and found a 10% average decline during 24 months after the Fukushima disaster. Nomura et al. [10] reported a 20% reduction in childbirths in 2012 compared to 2011 in Iwaki City, some 30 miles south of the Fukushima Daiichi nuclear power station (FDNPS). Hamamatsu et al. [9] analyzed the trend of LB in a so-called “disaster-stricken area” consisting of thirteen prefectures of the Kanto and the Tohoku region and determined the difference between observed and predicted numbers of LB in each month from December 2011 to June 2012. In five of the seven months, the numbers of observed births were significantly smaller than the predicted numbers. A closer look at the numbers of observed and predicted LB in Table 1 [9] reveals that, in December 2011, 3649 missing births were found, a 10.2% drop, more than in the subsequent three months combined with 3630 missing births (-3.6%). No significant decrease in LB in December 2011 was detected in their “non-disaster-stricken” area, i.e. the rest of Japan.

**Table 1:** Regression results with model (1) for the study region and areas A, B, C.

study region (deviance = 1071, df = 146)					
variable	O	E	O-E	(O-E)/E	p-value
pre	56915	58859.6	-1944.6	-3.3%	0.006
dec11	26660	29334.7	-2674.7	-9.1%	0.000
Q1	82224	83560.9	-1336.9	-1.6%	0.111
Q2	81948	85644.9	-3696.9	-4.3%	0.000
Q3	89822	90494.2	-672.2	-0.7%	0.443
Q4	86998	87429.3	-431.3	-0.5%	0.618
Area A (deviance = 329, df = 146)					
variable	O	E	O-E	(O-E)/E	p-value
pre	11823	12278.0	-455.0	-3.7%	0.011
dec11	5279	6139.7	-860.7	-14.0%	0.000
Q1	17445	17670.9	-225.9	-1.3%	0.287
Q2	17135	18106.1	-971.1	-5.4%	0.000
Q3	18802	18961.9	-159.9	-0.8%	0.472
Q4	17964	18100.8	-136.8	-0.8%	0.531
Area B (deviance = 945, df = 146)					
pre	45092	46578.7	-1486.7	-3.2%	0.012
dec11	21381	23193.9	-1812.9	-7.8%	0.000
Q1	64779	65889.1	-1110.1	-1.7%	0.113
Q2	64813	67537.8	-2724.8	-4.0%	0.000
Q3	71020	71531.2	-511.2	-0.7%	0.484
Q4	69034	69327.2	-293.2	-0.4%	0.685
Area C (deviance = 1771, df = 146)					
pre	116932	118336.2	-1404.2	-1.2%	0.271
dec11	58583	59364.1	-781.1	-1.3%	0.382
Q1	170674	170226.8	447.2	+0.3%	0.777
Q2	169062	170777.5	-1715.5	-1.0%	0.266
Q3	181379	181695.0	-316.0	-0.2%	0.843
Q4	175124	175363.6	-239.6	-0.1%	0.879

O, E: Observed and expected numbers of LB

The objective of the present study was to analyze the Japanese data of LB using a similar approach as that in Hamamatsu et al. 2014 [9] but with a focus on December 2011. After the triple meltdown at Fukushima Daiichi nuclear power plant (FDNPP) on March 12–15, 2011, the ambient dose rate at Fukushima Health Office jumped within hours to values above 20 μSv/h, more than 200-times above normal (see S1 Fig). According to ICRP Publication 90 [12], a high lethality risk for the conceptus exists during the pre-implantation period (first 8 days after conception). The high radiation exposure in the second half of March 2011 may have caused spontaneous abortions, manifesting as a decrease in LB nine months later.

## Data and methods

The Japanese Statistics Bureau publishes demographical information compiled by the Ministry of Health, Labor, and Welfare. Monthly numbers of LB from Japan, 2002 through 2019, are available online on a prefecture-level [13]. For the present study, a study region was chosen, consisting of Fukushima Prefecture and eleven nearby prefectures (Miyagi, Gunma, Tochigi, Ibaraki, Iwate, Akita, Yamagata, Niigata, Saitama, Tokyo, and Chiba). It differs slightly from the “disaster-stricken” area selected by Hamamatsu et al. [9] which included the prefectures Aomori in the north of Tohoku region and Kanawaga Prefecture to the south of Tokyo but did not include Niigata Prefecture which is bordering on Fukushima Prefecture.

In Hamamatsu et al. [9], a time window from December 2011 through June 2012 was used to check the data for a possible impact of the Fukushima accident on LB because data was only available until June 2012. For each of the seven months of the time window, the predicted (E) number of LB was estimated from the trend of LB in the same month in the years before the Fukushima accident, Jan 1997 through June 2011. A quadratic time trend was used for the regressions because, according to the authors of [9], it fitted the data best. Whenever the observed (O) number of births was greater or less than the limits of the 95% confidence interval of E, the difference between O and E was considered statistically significant.

In the present study, I applied a different method to determine any effects of the Fukushima accident on LB. To allow for monthly variation, I used dummy variables for February (*feb*) through December (*dec*) with January as the reference month. In leap years (2004, 2008, 2012, 2016), the numbers of LB in February were multiplied by 28/29. As a study period, I used the data from Jan 2006 to Dec 2019 with a time window from October 2011 to December 2012 for checking any effects of the Fukushima disaster on live births. October 2011 was chosen as the beginning of the time window to allow for possible radiation effects on the embryo, and December 2012 as its end assuming that any effect of the Fukushima accident would not last more than one year. The time window was divided into three periods according to time of conception before (October-November 2011, dummy variable *pre*), during (December 2011, *dec*11), and after (2012) the Fukushima accident in March 2011. Dummy variables *Q*1, *Q*2, *Q*3, *Q*4 tagged the four quarters of 2012.

Poisson regression with a linear (*t*) and a quadratic (*t*2) time variable was applied, where time *t* was defined as the beginning of a calendar year minus 2000. Regression model (1) has the following form:

log(LB(t))∼intercept+t+t2+feb+⋯+dec+pre+dec11+Q1+Q2+Q3+Q4 (1)

Here, *log*(*LB*(*t*)) is the natural logarithm of live births, and the tilde symbol (~) indicates that *log*(*LB*(*t*)) is approximated by the formula after the tilde.

Since babies born in January 2012 and afterward were conceived after the Fukushima accident in March 2011, they were not exposed to the radiation bursts following the triple meltdown. Any effects on LB in 2012 are therefore likely due to stress after the earthquake and tsunami and fear of possible adverse radiation effects. To allow for a stepwise recovery of birth numbers in the first nine months of 2012, variable *post* was defined as *post* = 1 in the first quarter, *post* = 2/3 in the second quarter, *post* = 1/3 in the third quarter, and *post* = 0 in the fourth quarter. This stepwise recovery was successfully applied by the present author in an analysis of live births from the City of Kiyv after the Chernobyl accident (see S2 Fig). Model (2) has the following form:

log(LB(t))∼intercept+t+t2+feb+⋯+dec+pre+dec11+post+Q2+Q3+Q4 (2)

Mind that model (2) is just another form of model (1) where variables *Q*2, *Q*3, *Q*4 estimate any deviation in quarters Q2, Q3, Q4 from the regression model instead of the deviation from the undisturbed long-term trend.

### Dose dependency

**Fig 1:**
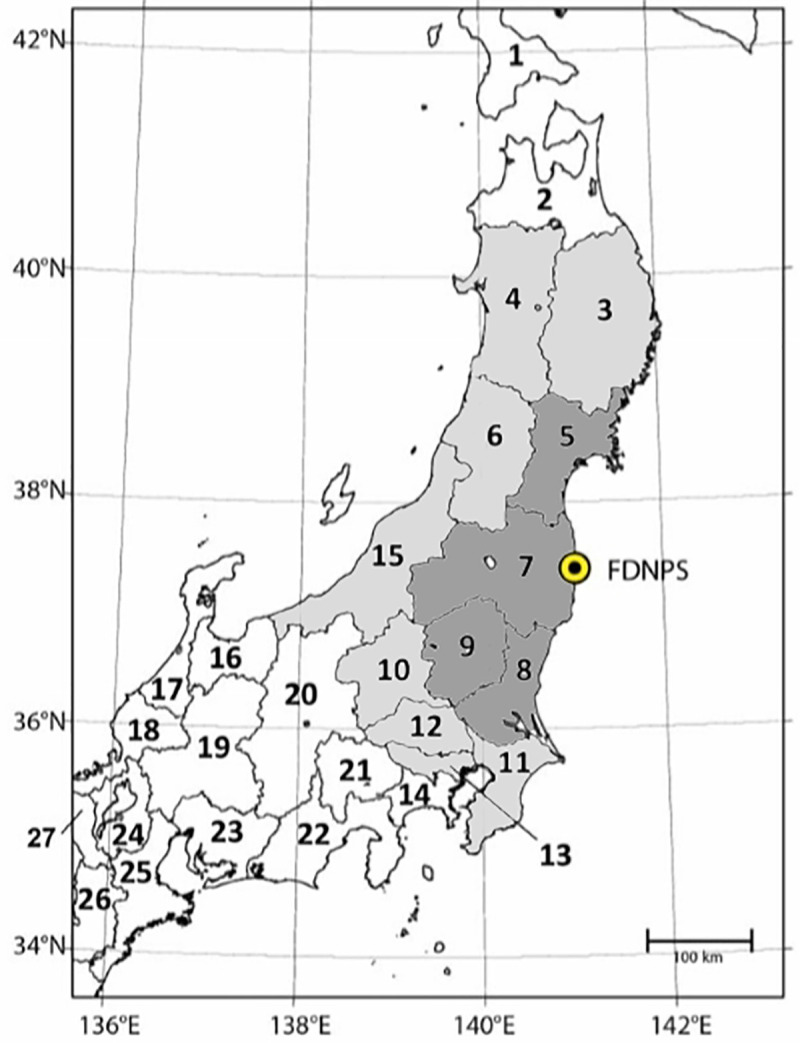
Map of prefectures around Fukushima Daiichi Nuclear Power Station (FDNPS). Area A (shaded dark grey): Prefectures Fukushima (7), Miyagi (5), Tochigi (9), Ibaraki (8); Area B (shaded light grey): Prefectures Iwate (3), Akita (4), Yamagata (6), Niigata (15), Gunma (10), Saitama (12), Tokyo (13), Chiba (11).

To check for a possible dose-dependency of the effects in Oct/Nov 2011 and in Dec 2011, the study region was divided into two areas of different mean radiation dose: (a) Fukushima plus three adjacent prefectures: Miyagi, Tochigi, and Ibaraki (Area A), and (b) eight surrounding prefectures: Iwate and Akita to the north, Yamagata and Niigata to the west; and Gunma, Saitama, Tokyo, and Chiba to the south (Area B). Japan minus the study region served as the comparison region (Area C). A map of the prefectures is shown in Fig 1. Mean effective doses to adults in the first year after the accident are given in Table 5 of the UNSCEAR 2013 report Annex A [14] for three residential areas: Fukushima prefecture (group 2), 1.0–4.3 mSv; prefectures Miyagi, Ibaraki, Gunma, Tochigi, Iwate, and Chiba (group 3), 0.2–1.4 mSv; and the rest of Japan (group 4), 0.1–0.3 mSv. For the present study, mean dose values of 1, 0.5, and 0.1 mSv were assumed in areas A, B, and C, respectively.

The combined regression model uses common parameters for monthly variation but allows for individual trend parameters (*intercept*, *t*1, *t*2) and individual effect sizes in Oct/Nov 2011, Dec 2011, and the 4 quarters of 2012, denoted by the dummy variables *preA*, *preB*, *preC*, *decA*, *decB*, *decC*, *Q*1*A*, *Q*1*B*, *Q*1*C*,…,*Q*4*A*, *Q*4*B*, *Q*4*C*. Model (3) has the following form:

log(LB(t))∼intercept+A+B+t+t2+t:A+t2:A+t:B+t2:B+feb+mar+⋯+dec+preA+preB+preC+decA+decB+decC+Q1A+Q1B+Q1C+⋯+Q4A+Q4B+Q4C (3)

Here, *t*: *A*, *t*2: *A*, *t*: *B*, *t*2: *B* are interactions that allow for differences in the estimates of *t* and *t*2 in areas A and B versus Area C.

To estimate the dose-dependency of the effects in Oct/Nov 2011 and Dec 2011, the expressions *preA*+*preB*+*preC* and *decA*+*decB*+*decC* in model (3) are replaced in model (4) by *pre*+*pre*: *dose* and *dec*11+*dec*11: *dose*, where the interactions *pre*: *dose* and *dec*11: *dose* allow for a possible dose-dependency of variables *pre* and *dec*11. As in model (2), variables *Q*1, *Q*2, *Q*3, *Q*4 are replaced by a single variable *post*, i.e. variables *Q*1*A*, *Q*2*A*, *Q*3*A*, *Q*4*A* are replaced by variable *postA* and so forth. As in model (2), variables *Q*2, *Q*3, *Q*4 estimate deviations of the observed live births from the expected trend and *Q*2: *dose*, *Q*3: *dose*, *Q*4: *dose* allow for possible dose-dependencies of the effects in quarters *Q*2, *Q*3, *Q*4. Model (4) has the following form:

log(LB(t))∼intercept+t+t2+A+B+t:A+t2:A+t:B+t2:B+feb+⋯+dec+pre+pre:dose+dec11+dec11:dose+postA+postB+postC+Q2+Q2:dose+Q3+Q3:dose+Q4+Q4:dose (4)

Statistical software R (www.r-project.org) [15] was used for data analysis and plotting. To adjust for overdispersion, the function glm(), family = quasipoisson was applied. The quasi-Poisson model uses the mean regression function and the variance function from the Poisson GLM package but leaves the dispersion parameter unrestricted. Two-sided t-tests were used and a p-value of p *<* 0.05 was considered statistically significant. The dispersion parameter (OD, for “overdispersion”), defined as the residual deviance (dev) divided by the degrees of freedom (df), was chosen as a measure of the goodness of fit.

## Results

In their analysis of live births from Japan, Hamamatsu et al. [9] used a linear-quadratic trend because, according to [9], “it best fitted the longitudinal trend during the pre-disaster-impact period in Japan”. To check this assertion, I conducted a Poisson regression with a linear-quadratic time trend of the annual birth numbers from 1997 through 2011, i.e. the period considered in [9] as “pre-disaster-impact period”. The data exhibited very large over-dispersion; the residual deviance (dev) was dev = 3375 with 12 degrees of freedom (df). The dispersion parameter OD = dev/df was 281. A fourth-degree polynomial fits the data much better (dev = 1755, df = 10, p = 0.002) than a second degree polynomial. Furthermore, there is a significant drop in 2005 (p = 0.002).

**Fig 2:**
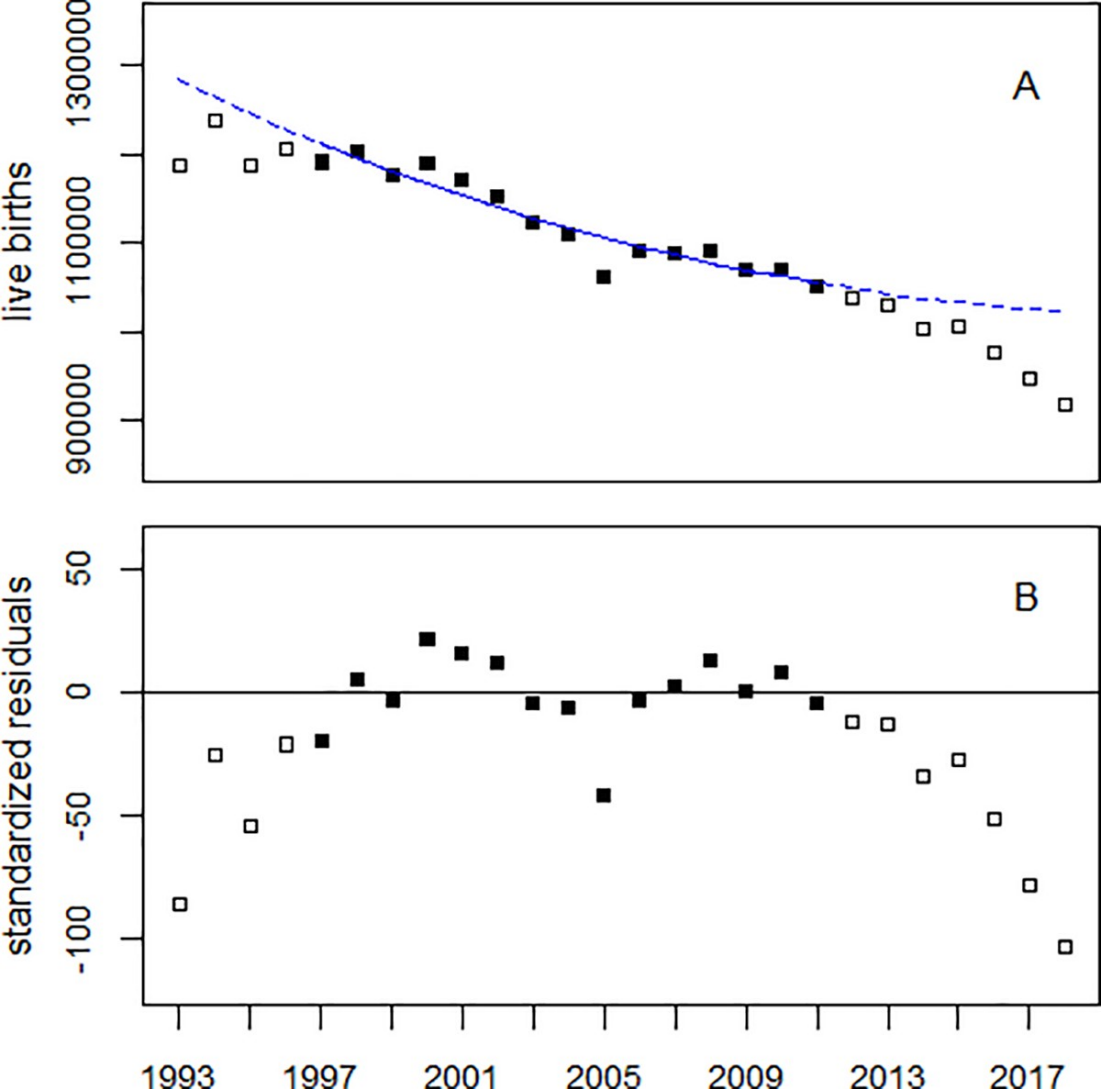
Panel A: Monthly numbers of LB from Japan, 1993–2018, and result of a Poisson regression of the data from 1997 to 2011 (black dots) with a linear-quadratic time trend. The broken line shows the extrapolated trend line. Panel B: Deviations of the observed numbers from the predicted trend in units of standard deviations (standardized residuals).

Fig 2 displays annual live births from Japan, 1993–2018. The solid line represents the regression result, and the broken line shows the extrapolation of the trend until 2018. After 2011, the observed numbers of live births deviate from the predicted trend, see the plot of residuals (panel B). Thus, the linear-quadratic model leads to a systematic overestimation of the birth deficit after 2011.

Next, a regression of the monthly data from the study region, 2006–2019, was conducted with model (1). The residual deviance was 1440 (df = 148). A third-degree polynomial for the time trend resulted in a significant improvement of the fit (dev = 1165, p < 0.0001). The model fit improved again with a fourth-degree polynomial (dev = 1071, p = 0.0005), but not with a fifth-degree polynomial (p = 0.84). Thus, a fourth-degree polynomial for the time trend was henceforth used in all analyses.

The results of a regression of live births from the study regions with model (1) are listed in Table 1. In parentheses, the deviance und the degrees of freedom are included in the headline. Significant reductions in LB were found in Oct/Nov 2011 (-3.3% (95% CI: -5.6%, -1.0%), p *=* 0.006), in December 2011 (-9.1 (-12.2, -6.0)%, p < 0.001), in the first quarter (Q1) of 2012 (-1.6 (-3.5, +0.4)%, p = 0.11) and in the second quarter (Q2) (-4.3 (-6.2, -2.4)%, p < 0.001), but not in the third (Q3) (-0.7%, p = 0.44) and fourth (Q4) quarter (-0.5%, p = 0.62). The percent reduction in LB was more than 5-times greater in Dec 2011 than in Q1 2012.

Regressions with model (1) were also carried out for the two sub-areas of the study region (areas A and B) and the rest of Japan (Area C). The regression results are included in Table 1. In December 2011, the decrease was greater in Area A with -14.0 (-17.6, -10.3) % than in Area B with -7.8 (-11.1, -4.5) %, while no notable decrease was found in Area C with -1.3 (-4.2, +1.7) %, p = 0.38.

**Fig 3:**
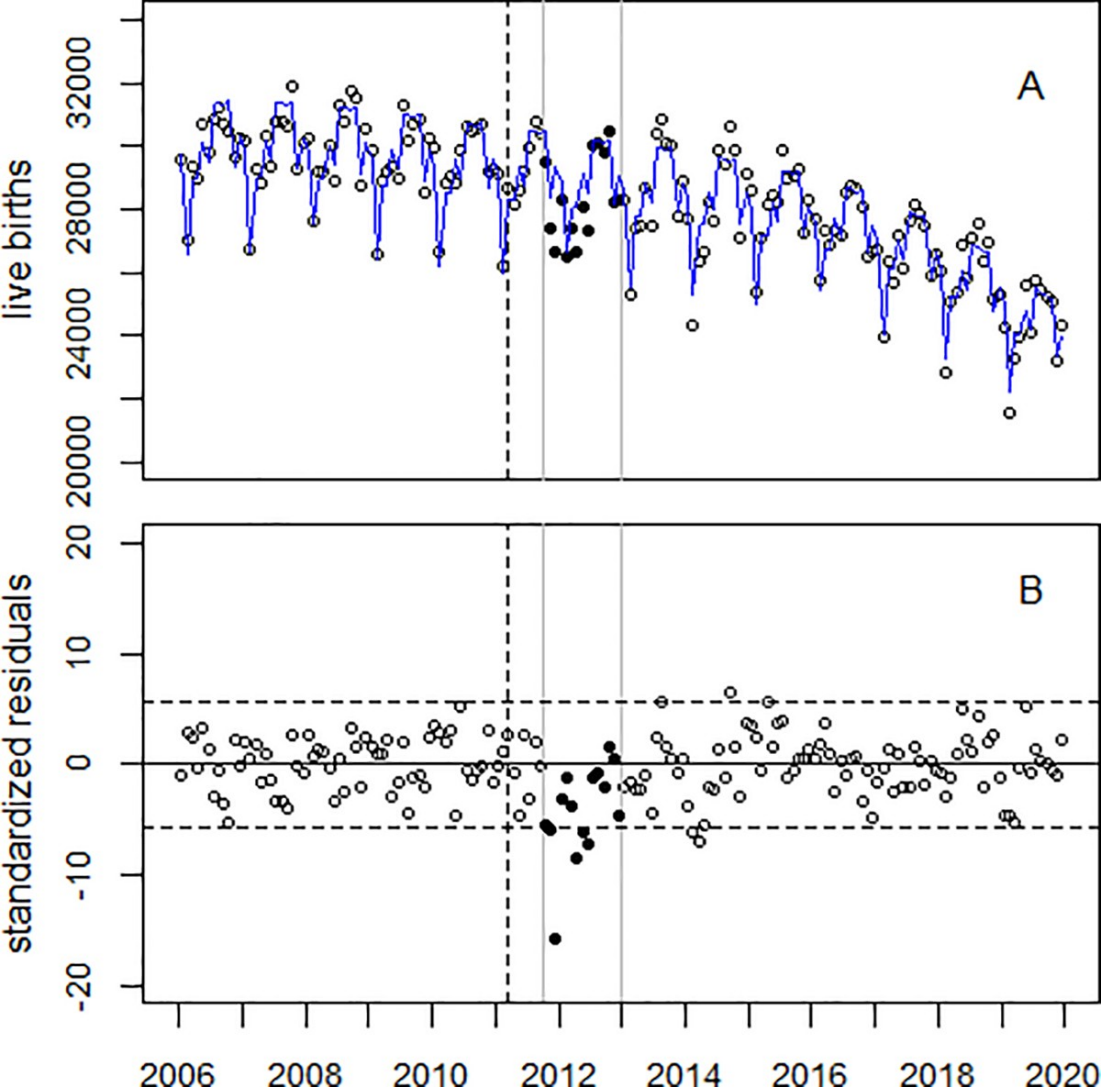
Panel A: Monthly numbers of LB in the study region, 2006–2019, and trend line. Panel B: Deviations of the observed numbers from the expected trend in units of standard deviations (standardized residuals). The black circles show the data in the time window (Oct 2011 through Dec 2012); the broken vertical line denotes March 2011.

**Fig 4:**
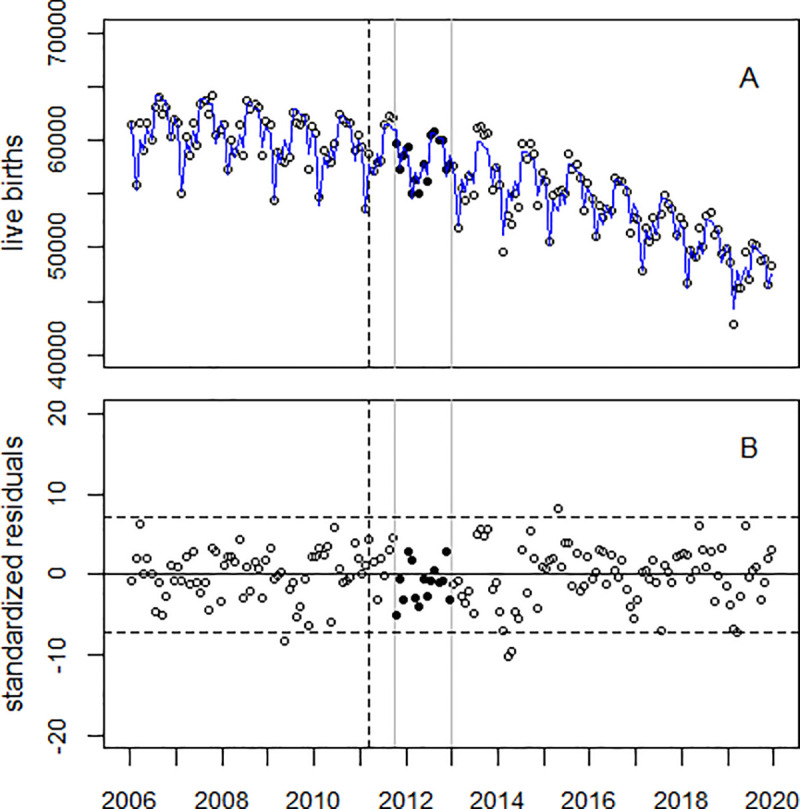
Panel A: Monthly numbers of LB in Japan minus the study region (Area C), 2006–2019, and trend line. Panel B: Standardized residuals. The black circles show the data in the time window (Oct 2011 through Dec 2012); the broken vertical line denotes March 2011.

Fig 3 upper panel shows the trend of LB in the study region and the regression line; the lower panel displays the residuals. The trend of LB in the comparison region (Japan minus the study region) is shown in Fig 4. The corresponding figures for areas A and B are provided in S3 and S4 Figs.

Next, a regression of the data from the study region with model (2) was conducted which allows for a stepwise decrease of the effect in the first three quarters of 2012. The results, i.e. variable names, parameter estimates, standard errors of parameter estimates (SE), t-values, and p-values, are listed in Table 2. Here, variables *Q*2, *Q*3, and *Q*4 estimate the deviations between the observed numbers of LB in quarters Q2, Q3, Q4, and the numbers expected from the regression model, whereas in model (1), variables *Q*2, *Q*3, *Q*4 estimate the deviations from the long term trend. As an example, variable *Q*2 in model (1) equals *post*+*Q*2 in model (2) and so forth. The effect of variable *Q*2 was statistically significant (-0.033 ± 0.012, p = 0.006) while the estimates of *Q*3 and *Q*4 did not differ notably from zero (p = 0.84 and p = 0.62, respectively).

**Table 2:** Regression results with model (2) for the study region and areas A, B, C.

Study region				
variable	estimate	SE	t-value	p-value
pre	-0.0336	0.0120	-2.791	0.006
dec11	-0.0956	0.0174	-5.509	0.000
post	-0.0162	0.0101	-1.602	0.111
Q2	-0.0333	0.0118	-2.814	0.006
Q3	-0.0020	0.0101	-0.203	0.839
Q4	-0.0049	0.0099	-0.500	0.618
Area A				
pre	-0.0378	0.0147	-2.576	0.011
dec11	-0.1510	0.0216	-6.993	0.000
post	-0.0131	0.0122	-1.069	0.287
Q2	-0.0464	0.0143	-3.240	0.001
Q3	-0.0041	0.0122	-0.337	0.736
Q4	-0.0076	0.0121	-0.628	0.531
Area B				
pre	-0.0324	0.0127	-2.554	0.012
dec11	-0.0814	0.0182	-4.469	0.000
post	-0.0171	0.0107	-1.593	0.113
Q2	-0.0298	0.0125	-2.383	0.018
Q3	-0.0015	0.0106	-0.139	0.889
Q4	-0.0042	0.0104	-0.406	0.685
Area C				
pre	-0.0119	0.0108	-1.104	0.271
dec11	-0.0132	0.0151	-0.877	0.382
post	0.0026	0.0090	0.283	0.777
Q2	-0.0118	0.0106	-1.115	0.267
Q3	-0.0026	0.0091	-0.285	0.776
Q4	-0.0014	0.0090	-0.152	0.879

^a^ SE: Standard error of estimate

The regression results for Areas A, B, and C are also listed in Table 2. The effect of variable *Q*2 was greater in Area A (-0.046 ± 0.014, p = 0.001) than in Area B (-0.030 ± 0.013, p = 0.018) and Area C (-0.012 ± 0.011, p = 0.27). Again, the effects of *Q*3 and *Q*4 didn’t differ notably from zero in all three areas A, B, C. This means that the assumed stepwise reduction of the effect in 2012 was confirmed except for the drop in Q2.

### Combined regression

The combined regression of the data from areas A, B, C with model (3) yielded dev = 3135 (df = 424); the dispersion parameter was OD = 7.39. The drop in December 2011 was highly significant in Areas A and B; the estimates of variables *decA* and *decB* were -0.160 ± 0.038 and -0.082 ± 0.019, respectively. The effect of *decC* was not statistically significant (-0.011 ± 0.012, p = 0.34). Significant effects were detected in Area B, quarters Q1, Q2. The regression results with model (3) for all variables in the time window are listed in Table 3.

**Table 3:** Results of combined regression with model (3).

variable	estimate	SE^a^	t-value	p-value
preA	-0.0480	0.0253	-1.896	0.059
preB	-0.0271	0.0131	-2.080	0.038
preC	-0.0130	0.0082	-1.580	0.115
decA	-0.1615	0.0373	-4.324	0.000
decB	-0.0819	0.0187	-4.381	0.000
decC	-0.0120	0.0115	-1.041	0.298
postA	-0.0166	0.0212	-0.780	0.436
postB	-0.0256	0.0111	-2.312	0.021
postC	0.0063	0.0069	0.906	0.365
Q2A	-0.0323	0.0247	-1.307	0.192
Q2B	-0.0196	0.0128	-1.526	0.128
Q2C	-0.0173	0.0080	-2.154	0.032
Q3A	-0.0033	0.0211	-0.155	0.877
Q3B	0.0024	0.0109	0.220	0.826
Q3C	-0.0042	0.0069	-0.608	0.543
Q4A	-0.0180	0.0209	-0.863	0.389
Q4B	-0.0007	0.0107	-0.065	0.948
Q4C	-0.0017	0.0068	-0.248	0.804
	deviance = 3295, df = 460, OD = 7.16			

^a^ SE: Standard error of estimate

The results of the regression with model (4), which allows for a dose-dependency of the variables *pre*, *dec*11, *Q*2, *Q*3, *Q*4, are listed in Table 4. The only significant dose-dependency was found for the effect in Dec 2011; the estimate of *dec*11: *dose* was -0.170 ± 0.037 per mSv, p < 0.001. The results for all other variables were not statistically significant.

**Table 4:** Results of combined regression with model (4).

variable	estimate	SE^a^	t-value	p-value
pre	-0.0091	0.0095	-0.958	0.339
pre:dose	-0.0377	0.0249	-1.513	0.131
dec11	0.0046	0.0133	0.348	0.728
dose:dec11	-0.1695	0.0360	-4.708	0.000
postA	-0.0185	0.0198	-0.932	0.352
postB	-0.0244	0.0101	-2.423	0.016
postC	0.0060	0.0068	0.883	0.378
Q2	-0.0156	0.0093	-1.681	0.093
dose:Q2	-0.0128	0.0243	-0.525	0.600
Q3	-0.0041	0.0080	-0.519	0.604
dose:Q3	0.0064	0.0208	0.308	0.758
Q4	0.0004	0.0078	0.046	0.963
dose:Q4	-0.0109	0.0205	-0.532	0.595
	deviance = 3299, df = 465, OD = 7.10			

^a^ SE: Standard error of estimate

To reduce the number of parameters, variables that had no notable effect on the goodness of fit were removed from the regression model. First, variables *Q*3, *Q*3: *dose*, *Q*4, *Q*4, *dose*, with associated p-values > 0.5, were eliminated. The deviance increased from 3299 to 3005 but the dispersion parameter reduced to 7.05. In the next step, variable *dec*11 (with a p-value of 0.71) was eliminated, which means that the linear association with dose was replaced by proportionality to dose in December 2011. As there is a strong correlation between intercept and slope, the linear relationship between dose and effect was retained for the effects in Oct/Nov 2011 and Q2 2012. The final model (5) had the following form:

log(LB(t))∼intercept+t+t2+t3+t4+A+B+t:A+t2:A+t:B+t2:B+t3:A+t3:B+t4:A+t4:B+feb+⋯+dec+pre+pre:dose+dec11:dose+postA+postB+postC+Q2+Q2:dose (5)

The regression with model (5) yielded deviance = 3306 (df = 470); the dispersion parameter was OD = 7.03. The results for the eight remaining variables in the time window are listed in Table 5. Significant effects are found for the dose dependencies in Oct/Nov 2011 (p = 0.002) and December 2011 (p < 0.001). The effect of variable *post* is greater in Area B (-2.4%. p = 0.014) than in Area A (-1.7%. p = 0.38), but there is no decrease in live births in Area C in 2012 except for a significant decrease in the second quarter of 2012 (-1.7%, p = 0.032; see Table 3).

**Table 5:** Results of combined regression with model (5).

variable	estimate	SE^a^	t-value	p-value
pre	-0.0089	0.0094	-0.947	0.344
pre:dose	-0.0369	0.0247	-1.495	0.135
dose:dec11	-0.1597	0.0257	-6.211	0.000
postA	-0.0169	0.0192	-0.878	0.381
postB	-0.0243	0.0098	-2.476	0.014
postC	0.0050	0.0065	0.762	0.446
Q2	-0.0147	0.0090	-1.622	0.105
dose:Q2	-0.0140	0.0237	-0.589	0.556
	deviance = 3306, df = 470, OD = 7.03			

An F-test was applied to estimate the combined effect of variables *pre*+*pre*: *dose* and *Q*2+*Q*2: *dose*. Deviance dev1 = 3306 (df1 = 470) obtained with model (5) was compared with deviance dev0 = 3377 (df0 = 472) resulting from a regression without *pre*+*pre*: *dose*. The F-test yielded *F* = 5.03, p = 0.007, so the effect in Oct/Nov 2011 was statistically significant. Deviance dev0 = 3364 (df0 = 472) resulted from a regression without *Q*2+*Q*2: *dose*. The F-test yielded *F* = 4.18, p = 0.016, so the drop in Q2/2012 significant.

The combined effect of the eight variables of the time window was highly statistically significant when compared with a regression without these variables (dev0 = 3796, df0 = 478; dev1 = 3306, df1 = 470, p = 4E-11, F test with 8 and 470 degrees of freedom).

**Fig 5:**
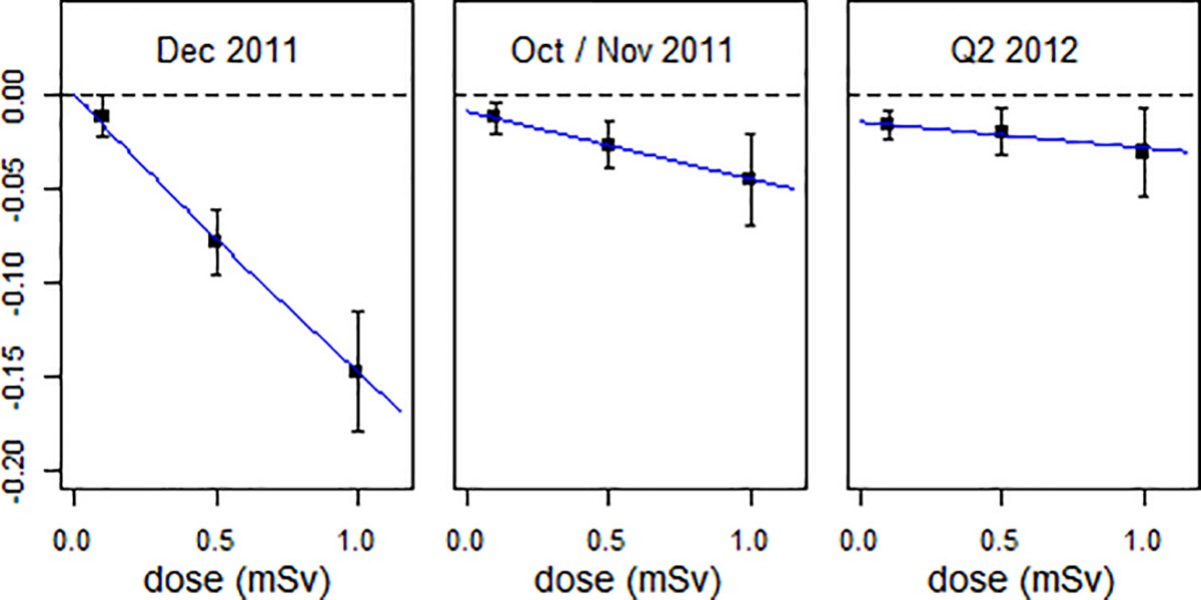
Reduction in live births in Dec 2011, Oct/Nov 2011, and Q2 2012 as a function of estimated mean effective dose. The error bars show standard errors of the estimate.

The dose dependencies of the effects in December 2011, Oct/Nov 2011, and Q2/2012 are presented in Fig 5.

#### Sensitivity analyses

To check whether the choice of mean doses had a notable effect on the results, regressions with model (5) were conducted with a fifty percent higher and lower mean dose in Area A. The residual deviance increased from 3306 (for 1 mSv in Area A) to 3317 (for 1.5 mSv), and the estimate of the dose-dependency in December 2011 (*dec*11: *dose*) changed from -0.160 ± 0.026 per mSv to -0.124 ± 0.020 per mSv. With a dose of 1/1.5 = 0.67 mSv in Area A, the deviance increased to 3318, and the estimate of *dec*11: *dose* changed to -0.183 ± 0.030 per mSv. Thus, the significance of the effect in December 2011 did not depend notably on the ratio of doses in Area A and Area B. A mean dose of 1 mSv yielded a better fit to the data than mean doses of 1.5 mSv or 0.67 mSv.

One might speculate that the drop in live births in December 2011 was a consequence of the earthquake and tsunami rather than a radiation effect. But then the effect in December 2011 should be greater in coastal prefectures than in inland prefectures. Analyses with model (1) on a prefecture-level showed that the drop in LB in December 2011 in the combined coastal prefectures Miyagi and Ibaraki was similar to the drop in the inland prefecture Tochigi; the effects of variable *dec*11 were -0.142 ± 0.025 and -0.133 ± 0.038, respectively.

Subsequently, a combined analysis with model (4) was conducted with prefectures regrouped according to UNSCEAR 2013, Annex A, Table 5. Group 2 in UNSCEAR Table 5 refers to Fukushima Prefecture; Group 3 refers to the six nearby prefectures Iwate, Miyagi, Ibaraki, Gunma, Tochigi, and Chiba; and Group 4 to the rest of Japan. Dose ranges are given in Table 5 for the three groups. From the upper and lower dose limits, I determined approximate mean doses of 2, 0.75, and 0.15 mSv in Groups 2, 3, and 4, respectively. The results of regressions with models (3) and (4) are available in S1 and S2 Tables. The estimates of the effects in December 2011 were -0.205 ± 0.080 (p = 0.011) in Fukushima Prefecture (Group 2), -0.110 ± 0.026 in Group 3 (p < 0.001), and -0.027 ± 0.010 in Group 4 (p = 0.006). The combined effect of variables *pre* and *pre*: *dose* was statistically significant (p = 0.005, F-test), as well as the combined effect of *Q*2 and *Q*2: *dose* (p = 0.011).

The dose dependencies of the reduction in LB in Dec 2011, Oct/Nov 2011, and Q2 2012 are shown in S5 Fig. The results are similar to those obtained with areas A, B, C defined in the present study.

## Discussion

In Japan, a highly significant 9% drop in live births (LB) was found in December 2011 in a study region consisting of Fukushima Prefecture plus ten surrounding prefectures. The number of missing births in December 2011 was greater than in the following three months combined. The effect was greater in the four prefectures with a higher mean radiation dose (Area A) than in the surrounding seven prefectures with a smaller mean dose (Area B). A significant reduction in LB was also found in Oct/Nov 2011 and the second quarter of 2012. A combined regression of the data from areas A, B, and C (rest of Japan) yielded a highly statistically significant association of the effect in December 2011 with the estimated mean doses in Areas A, B, C. A significant dose dependency was also found for the effect in Oct/Nov 2011.

The time lag of nine months between the massive drop in live births in Dec 2011 and the Fukushima disaster in March 2011 may indicate a possible adverse effect of the radiation spike on the conceptus after the triple meltdown on March 12–15.

The dose-dependent decrease in LB in Oct/Nov 2011 concerns births conceived before March 2011 and exposed to radiation blast in the critical period of embryonal development. In 2012, a decrease in LB was found in the study region but not in the comparison region. The effect decreased with time and was likely caused by the catastrophic situation in the aftermath of the earthquake and tsunami, and to public anxiety of possible adverse radiation effects. But there was an additional dose-dependent increase in the second quarter of 2012 which coincides with an increase in preterm births (less than 37 weeks) in Fukushima prefecture conceived 4–6 months after the Fukushima accident [16]. Perinatal mortality also exhibited a peak around April 2012 in Fukushima and four adjacent prefectures [17].

Next, the results of the present study are compared with those reported by Hamamatsu et al. in [9]. In Table 6, the observed (O) and predicted (E) numbers of live births in the study region are listed together with those for the disaster-stricken area in [9]. In both studies, the greatest drop in LB was found in Dec 2011 (-9.1% and -10.2%, resp.). Large decreases in LB were also detected in April (-5.1% and -7.7%, resp.) and June 2012 (-4.3% and 8.3%, resp.). For unknown reasons, the large 10% drop in LB in December 2011 was not mentioned by Hamamatsu et al. in the abstract nor result section.

**Table 6:** Observed (O) and predicted (E) numbers of live births in the present study and in [9].

	present study				Hamamatsu et al. [9]			
Month	O	E	O-E	(O-E)/E	O	E	O-E	(O-E)/E
Oct 2011	29522	30464.3	-942.3	-3.1%				
Nov 2011	27393	28395.3	-1002.3	-3.5%				
Dec 2011	26660	29334.7	-2674.7	-9.1%	32078	35727	-3649	-10.2%
Jan 2012	28332	28868.7	-536.7	-1.9%	32843	34753	-1910	-5.5%
Feb 2012	26487	26676.9	-189.9	-0.7%	30452	31097	-645	-2.1%
Mar 2012	27405	28015.3	-610.3	-2.2%	32689	33764	-1075	-3.2%
Apr 2012	26628	28053.9	-1425.9	-5.1%	31287	33905	-2618	-7.7%
May 2012	28033	29087.7	-1054.7	-3.6%	34033	34047	-14	0.0%
Jun 2012	27287	28503.2	-1216.2	-4.3%	32469	35409	-2940	-8.3%

The drop in LB in December 2011 complies with some of Bradford Hill’s criteria of causation [18]: (a) Strength of the association–the drop is highly statistically significant; (b) Temporality–it occurs nine months after the Fukushima accident; (c) Biological gradient—the effect is greater in higher contaminated prefectures (Area A) than in less contaminated prefectures (Area B); (d) Plausibility—according to ICRP Publication 90, “the dominant effect of pre-implantation irradiation is early death of the conceptus” [12, p.1].

### Strengths and limitations

The focus of this study was on the effect in December 2011, so the time window was divided into three periods according to the time of conception (before, during, and after March 2011). By splitting the study region into two areas of different mean distances from the FDNPS (as a proxy for dose), an association of the effect in December 2011with mean radiation dose could be established.

The main limitation of the study is that it is based on highly aggregated data; possible confounders could not be considered. The assumed values for the mean effective doses in the three areas of Japan are rough estimates and are not substantiated. Migration out of the study region may have influenced the results, but migration will mainly have occurred within the study region (e.g. from Area A to Area B).

To conclude, the present study found reduced live births in Oct 2011 through June 2012 in a study region consisting of Fukushima and ten neighboring prefectures. A prominent 9% drop was detected in December 2011, nine months after the Fukushima accident, which was greater in Fukushima and three adjacent prefectures than in the rest of the study region. The effect in December 2011 may be explained by the early death of the conceptus caused by the radiation burst after the triple meltdown at Fukushima Daiichi NPS on March 12–15, 2011.

## Supporting information

S1 File Monthly data of live births in areas A, B, C, 2006–2019. (TXT)

S1 Fig Ambient radiation levels (μSv/h) at different locations of Fukushima prefecture. (DOCX)

S2 Fig Trend of live births from Kiev-City and regression line. (DOCX)

S3 Fig Trend of live births and undisturbed trend line in Area A and standardized residuals. (DOCX)

S4 Fig Trend of live births and undisturbed trend line in Area B and standardized residuals. (DOCX)

S5 Fig Decrease in live births in the time window as a function of estimated mean dose. (DOCX)

S1 Table Regression results with model (3) in 3 regions of Japan defined in UNSCEAR 2013. (DOCX)

S2 Table Regression results with model (4) in 3 regions of Japan defined in UNSCEAR 2013. (DOCX)
