# Supplementary material for: Reduction in live births in Japan nine months after the Fukushima nuclear accident: An observational study
Source: PLoS One. 2021 Feb 25;16(2):e0242938. doi: 10.1371/journal.pone.0242938 (PMC7906319; doi:10.1371/journal.pone.0242938)
Supplement: S1 Fig — (DOCX) [file pone.0242938.s002.docx]

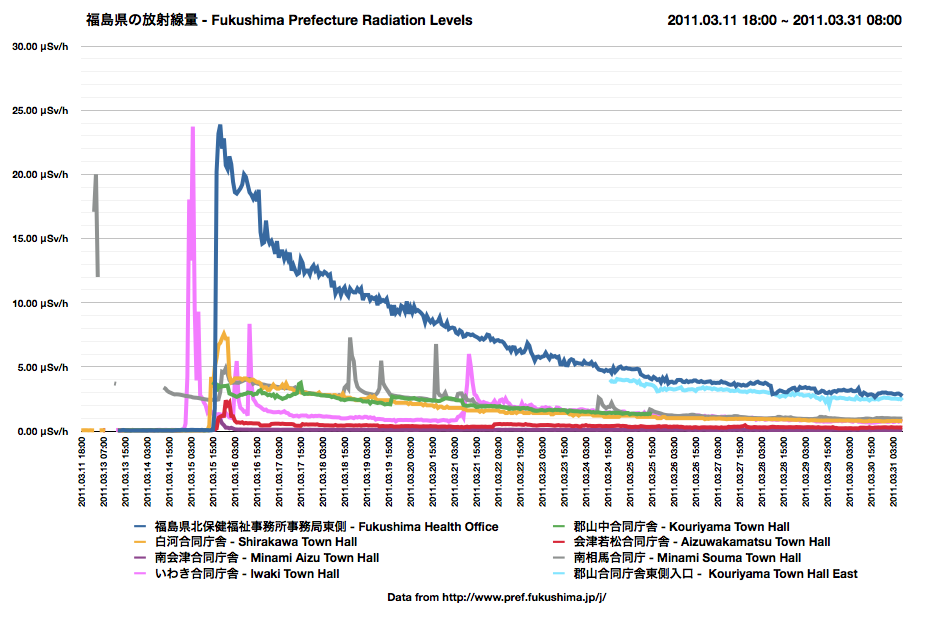


S1 Fig. Ambient radiation levels (µSv/h) at different locations of Fukushima prefecture.

Source:
https://commons.wikimedia.org/wiki/File:Fukushima_I_radiation,_Fukushima_Prefecture_2,_March_2011.png (accessed 12 Sept 2020)
