# Supplementary material for: Reduction in live births in Japan nine months after the Fukushima nuclear accident: An observational study
Source: PLoS One. 2021 Feb 25;16(2):e0242938. doi: 10.1371/journal.pone.0242938 (PMC7906319; doi:10.1371/journal.pone.0242938)
Supplement: S2 Fig — (DOCX) [file pone.0242938.s003.docx]

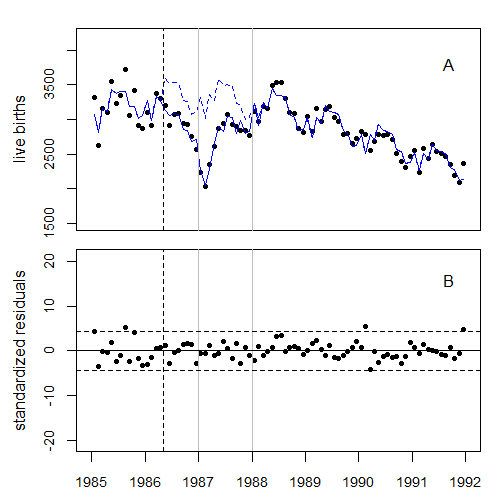


S2 Fig. Panel A: Trend of live births from Kiev-City and result of a Poisson regression with a third degree polynomial for the time trend and 11 variables for the monthly variations, i.e. for February through December with January as the reference month. The effect of the Chernobyl accident on live births is modelled by three dummy variables: (1) May-December 1986; (2) the first quarter (Q1) 1987; (3) variable post with post=1 in Q2, post=2/3 in Q3, post=1/3 in Q4 1987, and post=0 otherwise. Panel B shows the residuals in units of standard deviations (standardized residuals). As yet, this data is not published in the peer reviewed literature.
