# Supplementary material for: Reduction in live births in Japan nine months after the Fukushima nuclear accident: An observational study
Source: PLoS One. 2021 Feb 25;16(2):e0242938. doi: 10.1371/journal.pone.0242938 (PMC7906319; doi:10.1371/journal.pone.0242938)
Supplement: S3 Fig — (DOCX) [file pone.0242938.s004.docx]

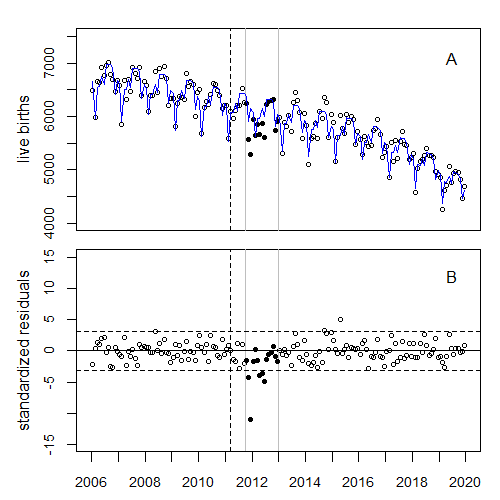


S3 Fig. Trend of live births and undisturbed trend line in Area A and standardized residuals

The black circles show the data in the time window (Oct 2011 through Dec 2012); the broken vertical line marks March 2011. The horizontal broken lines in Panel B indicate the range of 2 standard deviations, inflated by the square root of the dispersion parameter.
