# Supplementary material for: Reduction in live births in Japan nine months after the Fukushima nuclear accident: An observational study
Source: PLoS One. 2021 Feb 25;16(2):e0242938. doi: 10.1371/journal.pone.0242938 (PMC7906319; doi:10.1371/journal.pone.0242938)
Supplement: S5 Fig — (DOCX) [file pone.0242938.s006.docx]

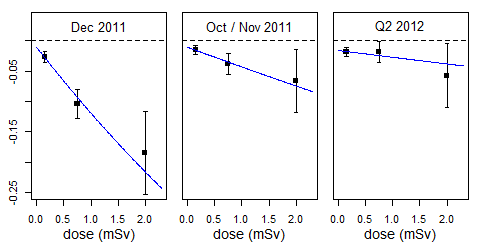


S5 Fig. Decrease in live births in the time window as a function of estimated mean dose.
Periods: December 2011 (Dec 2011), Oct-Nov 2011 (Oct/Nov 2011), and 2nd quarter of 2012 (Q2 2012). Error bars represent standard errors of the estimates.
