# Supplementary material for: Reduction in live births in Japan nine months after the Fukushima nuclear accident: An observational study
Source: PLoS One. 2021 Feb 25;16(2):e0242938. doi: 10.1371/journal.pone.0242938 (PMC7906319; doi:10.1371/journal.pone.0242938)
Supplement: S1 Table — (DOCX) [file pone.0242938.s007.docx]

S1 Table. Results of regression with model (3) in 3 regions of Japan defined in UNSCEAR 2013

Group 2: Fukushima Prefecture; 2 mSv mean dose in the first year
Group 3: Prefectures Iwate, Miyagi, Gunma, Tochigi, Ibaraki, Chiba; 0.75 mSv
Group 4: Rest of Japan, 0.15 mSv
Variables: pre: Oct/Nov 2011; dec11: Dec 2011; Q1, Q2, Q3, Q4: quarters of 2012

Regression model (3) (R notation):

m3 <- glm(LB*leap~A+B+t+t2+t3+t4+tA+tB+t2A+t2B+t3A+t3B+t4A+t4B+
 feb+mar+apr+may+jun+jul+aug+sep+oct+nov+dec+preA+preB+preC+decA+decB+decC+
 Q1A+Q1B+Q1C+Q2A+Q2B+Q2C+Q3A+Q3B+Q3C+Q4A+Q4B+Q4C,family=quasipoisson)

| variable | estimate | SE | t-value | p-value |
| --- | --- | --- | --- | --- |
| preA | -0.0687 | 0.0538 | -1.275 | 0.203 |
| preB | -0.0387 | 0.0181 | -2.143 | 0.033 |
| preC | -0.0154 | 0.0070 | -2.181 | 0.030 |
| decA | -0.2049 | 0.0804 | -2.548 | 0.011 |
| decB | -0.1102 | 0.0261 | -4.218 | 0.000 |
| decC | -0.0272 | 0.0099 | -2.742 | 0.006 |
| postA | -0.0942 | 0.0464 | -2.030 | 0.043 |
| postB | -0.0100 | 0.0152 | -0.660 | 0.510 |
| postC | -0.0010 | 0.0059 | -0.166 | 0.868 |
| Q2A | -0.0593 | 0.0541 | -1.095 | 0.274 |
| Q2B | -0.0181 | 0.0176 | -1.030 | 0.303 |
| Q2C | -0.0186 | 0.0069 | -2.684 | 0.008 |
| Q3A | -0.0302 | 0.0458 | -0.659 | 0.510 |
| Q3B | 0.0013 | 0.0151 | 0.086 | 0.931 |
| Q3C | -0.0026 | 0.0059 | -0.436 | 0.663 |
| Q4A | -0.1054 | 0.0459 | -2.295 | 0.022 |
| Q4B | -0.0046 | 0.0149 | -0.308 | 0.758 |
| Q4C | -0.0006 | 0.0058 | -0.104 | 0.917 |

Residual deviance = 3064 on 460 degrees of freedom
