# Supplementary material for: Reduction in live births in Japan nine months after the Fukushima nuclear accident: An observational study
Source: PLoS One. 2021 Feb 25;16(2):e0242938. doi: 10.1371/journal.pone.0242938 (PMC7906319; doi:10.1371/journal.pone.0242938)
Supplement: S2 Table — (DOCX) [file pone.0242938.s008.docx]

S2 Table. Results of regression with model (4) in 3 regions of Japan defined in UNSCEAR 2013

Regression model (4):

m4 <- glm(LB*leap~A+B+t+t2+t3+t4+tA+tB+t2A+t2B+t3A+t3B+t4A+t4B+
 feb+mar+apr+may+jun+jul+aug+sep+oct+nov+dec+pre+pre:dose+dec11+dec11:dose+
 postA+postB+postC+Q2+Q2:dose+Q3+Q3:dose+Q4+Q4:dose,family=quasipoisson)

| variable | estimate | SE | t-value | p-value |
| --- | --- | --- | --- | --- |
| pre | -0.0106 | 0.0086 | -1.240 | 0.216 |
| pre:dose | -0.0335 | 0.0221 | -1.512 | 0.131 |
| dec11 | -0.0108 | 0.0122 | -0.884 | 0.377 |
| dec11:dose | -0.1165 | 0.0326 | -3.572 | 0.000 |
| postA | -0.1037 | 0.0430 | -2.413 | 0.016 |
| postB | -0.0070 | 0.0140 | -0.503 | 0.615 |
| postC | -0.0013 | 0.0059 | -0.217 | 0.828 |
| Q2 | -0.0162 | 0.0084 | -1.919 | 0.056 |
| Q2:dose | -0.0114 | 0.0218 | -0.521 | 0.602 |
| Q3 | -0.0012 | 0.0072 | -0.166 | 0.869 |
| Q3:dose | -0.0051 | 0.0186 | -0.273 | 0.785 |
| Q4 | 0.0058 | 0.0071 | 0.822 | 0.412 |
| Q4:dose | -0.0334 | 0.0184 | -1.817 | 0.070 |

Residual deviance = 3084 on 465 degrees of freedom
